# Maternal dietary protein and amino acid intake is not associated with the amino acid composition of human milk in an affluent environment

**DOI:** 10.1017/S0007114524001600

**Published:** 2024-09-14

**Authors:** Hannah Juncker, Peiheng Wang, Inga Petersohn, Louise Naz West, Eva Naninck, Johannes van Goudoever, Elske Brouwer-Brolsma, Aniko Korosi

**Affiliations:** 1 Brain Plasticity group, Swammerdam Institute for Life Sciences, University of Amsterdam, Amsterdam, The Netherlands; 2 Amsterdam UMC, Emma Children’s Hospital, Amsterdam Reproduction and Development Institute, University of Amsterdam, Amsterdam, The Netherlands; 3 Division of Human Nutrition and Health, Wageningen University, Wageningen, The Netherlands; 4 Food Quality & Design Group, Wageningen University & Research, Wageningen, The Netherlands; 5 Laboratory of Organic Chemistry, Wageningen University & Research, Wageningen, The Netherlands

**Keywords:** Amino acid, Human milk, Diet, Lactation, Intake

## Abstract

Amino acids (AA) are essential nutrients in human milk (HM) and critical for infant growth and development. Several maternal lifestyle factors have been suggested to influence HM AA composition, with possible consequences for the breastfed infant. Whether maternal dietary protein and AA intake is associated with AA concentrations in HM is still largely unknown. Therefore, the aim of this study was to investigate the association between maternal dietary AA intake and AA concentrations in HM over the first month postpartum. Data from the observational longitudinal Amsterdam Mother’s Milk study were used, consisting of 123 lactating women in their first postpartum month. HM samples were collected three times, on day 10, 17 and 24 postpartum. Maternal dietary protein and AA intake on these collection days was assessed using three 24-h recalls. HM protein-bound and free AA (BAA and FAA, respectively) were analysed by liquid chromatography. Associations between maternal AA intake and AA concentrations in HM were assessed using linear mixed models. Maternal intake was negatively associated with milk concentrations of free arginine (–0·0003; *P* = 0·01) and free lysine (–0·0004; *P* = 0·03) and was positively associated with free glutamine (0·002; *P* = 0·03) and free threonine (0·0008; *P* = 0·03). However, these associations were attenuated after correction for multiple testing. Both the quality and quantity of dietary protein intake in lactating women do not seem to influence the amino composition of their breast milk when living in an affluent environment.

Human milk (HM) is an infant’s optimal source of nutrition in the first months after birth, affecting a child’s health throughout life. Next to providing the essential nutrients for healthy growth and development, HM has other health-related benefits for both mother and child^(1)^. The WHO recommends exclusive breastfeeding for the first 6 months of life^(2)^. Additionally, HM is recommended as an essential part of the diet until the child is 1 year old and a significant additional source of nutrition until 2 years of age^(2)^.

Amongst the essential nutrients associated with the positive effects of HM are the amino acids (AA)^(3)^. HM contains AA primarily as part of protein structures; moreover, free amino acids (FAA) account for 5–10 % of total AA in milk^(4,5)^. AA are the building blocks for healthy growth and development^(3)^. For example, protein-bound AA (BAA) improve digestion by increasing the uptake of other nutrients and play roles in transportation, enzymatic activity and immune system regulation^(6,7)^. While the importance of HM BAA in early nutrition is well established^(3,8)^, the role of FAA is less well known. FAA are more easily absorbed than BAA, play an essential role in the body’s nitrogen balance and are responsible for the initial change in plasma FAA after a meal^(9)^. It has been suggested that FAA have multiple bioactive roles as regulators of key pathways necessary for growth, development and immunity^(3)^.

HM AA concentrations are known to change over the course of lactation^(4)^. Under normal circumstances, HM BAA decreases, while total FAA increases over the course of lactation^(4)^. In addition, some other factors have been suggested to influence HM AA composition, including the time of day, gestational age at delivery and maternal genetic profile^(1,3)^. Changes in HM AA composition are assumed to align with infant needs. However, external factors, such as maternal smoking and lifestyle, may have an impact as well^(3,10)^. The impact of maternal dietary intake on HM AA composition is not yet understood.

Several important HM components have been shown to be influenced by the maternal diet^(11–13)^. For example, it is well accepted that dietary fatty acid intake influences the composition of HM^(14)^. However, similar studies for protein and AA intake show contradicting results and only focus on total protein intake instead of the intake of specific AA. Where four previous studies found that decreased total dietary protein intake was associated with lower protein and/or AA in HM^(15–18)^, four other studies found no such association^(19–22)^. Most of these studies are limited by relatively small sample sizes and have methodological restraints, for example, undetailed information on maternal dietary intake and varying stages of lactation. More importantly, none of the studies mentioned above investigated the maternal intake of specific AA and their relation with HM AA concentrations.

The first month postpartum is a sensitive time window in which breastfed infants are dependent on HM as their only source for nutrients. Therefore, knowledge of the factors influencing HM during these first weeks after birth is extremely important. Understanding if and how maternal dietary AA intake influences the HM AA composition is a starting point for advancing and optimising newborn and lactating mothers’ nutrition. Therefore, the aim of this research is to explore whether maternal protein/AA intake is associated with AA concentrations in HM over the first month after birth by studying them (1) as total BAA and total FAA, (2) as individual BAA and FAA, (3) as essential BAA/FAA and non-essential BAA/FAA separately and (4) based on their precursor group.

## Methods

### Research design and participant recruitment

This study is a secondary analysis of data from the Amsterdam Moedermelk Studie, a multicentre observational cohort study^(23,24)^. In the Amsterdam Moedermelk Studie, it was investigated if, and how, maternal stress affects HM composition. In this original study, three groups of mothers were included: (1) a control group, consisting of mothers who gave birth at term to a healthy infant; (2) a term high stress group, consisting of mothers who gave birth at term to an infant who was hospitalised after birth; and (3) a preterm high stress group, consisting of mothers who gave birth preterm to an infant who was hospitalised after birth. In the present study, where we investigate the association between dietary AA/protein intake and HM AA composition, all mothers out of the three study groups were included.

Participants were recruited during pregnancy or within the first 10 d after giving birth, via social media, flyers at midwife practices or in the maternal or neonatal ward. Mothers were eligible to participate when they were 18 years of age or older and if they had the intention to breastfeed their infant for at least the first month after birth. Exclusion criteria were maternal (gestational) diabetes mellitus, maternal use of psychopharmaceuticals or glucocorticoid medication, major congenital disease of the neonate, a life expectancy of the neonate of less than 1 month or no data available on maternal dietary intake. Inclusion took place between November 2017 and December 2019. Written informed consent was obtained from all participants prior to participation. Data were handled anonymously. This study was conducted according to the guidelines laid down in the Declaration of Helsinki, and all procedures involving human subjects were approved by the Ethics Committee (METC AMC) on the 2nd of May 2017.

### Study timeline

**Fig. 1.**
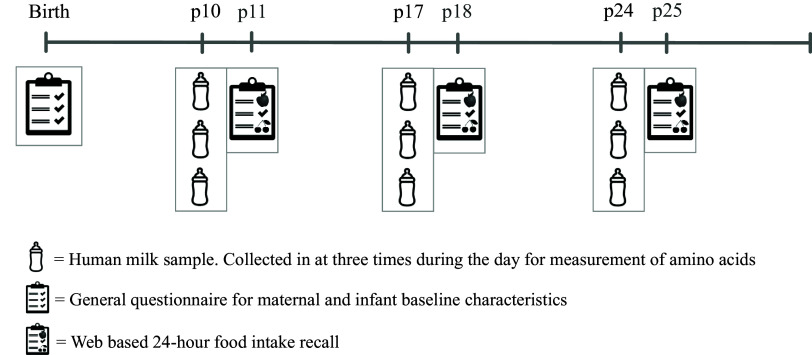
Study timeline. p = postpartum day.

Inclusion occurred between the day of birth and day 10 after birth. At inclusion, participants filled out a questionnaire on their personal characteristics, general health and pregnancy. Following that, the study comprised of three collection days; these days were on postpartum day (p) 10, 17 and 24. On these collection days, participants collected three HM samples, preferably in the morning, afternoon and evening. The day after the collection day, participants filled out a 24-h recall (24-hR) to collect information on their exact food intake on the collection day. The study timeline is presented in Fig. 1.

### Milk sample collection

At every collection day (p10, p17 and p24), participants collected three HM samples to measure the concentrations of BAA and FAA: a morning, afternoon and evening sample. To make sure the circadian variation in HM AA was represented in the samples^(25,26)^, the three samples were equally mixed into one sample. To make sure the HM sample would contain a mixture of foremilk and hindmilk, participants were requested to fully empty one breast before feeding their infant, mix the milk and thereafter donate 5 ml of HM in a sterile container (Sarstedt, Germany). Participants were free to choose from which breast the milk was collected. Participants were requested to write down the date and time of milk collection, the way of pumping (i.e. manually or electric pump) and the total amount of milk that was collected. Participants stored the milk samples in their freezer (–20°C) up until collection by the researcher. At the study site, HM samples were stored at –20°C until analysis.

### Dietary assessment

Dietary intake was quantified using three 24-hR days completed through the web-based self-administered 24-hR tool called Compl-eat™^(27,28)^. Compl-eat™ has been validated in an adult population, with stratified data for men and women^(27)^. Within the Compl-eat™ tool, participants were invited via an email, sent at 07.00 h the day after HM collection, to report their previous day’s intake from waking up until waking up the next morning. Compl-eat was accessible until midnight the same day. Before the participants reported their intake, they received access to two instruction videos explaining how to complete the quick list and details (type and amount) of the foods consumed. Compl-eat allowed participants to select foods and standard recipes commonly used by the Dutch population^(25)^. Portion sizes were provided in standard portions, weight in grams, using regularly used household measures^(26)^. Participants could also use a recipe module to report their intake of a certain dish by choosing or adapting a standard recipe, listing all the ingredients in their own recipe and indicating the proportion of the dish consumed. Yield and retention factors were automatically taken into account where appropriate. Compl-eat also allowed participants to record notes for clarification, for example, a description of a food that the participant could not find on the food list. Once the recall was completed, participants were prompted to record commonly forgotten foods such as sugar in coffee, snacks, fruit and cooking fat. Trained dietitians checked all the web-based 24 hR for their completeness and unusual portion sizes and processed all notes made by the participants. Participants were not contacted for clarifications. Errors and notes were adjusted in a standardised way, using standard portion sizes and recipes according to a protocol, for example, a report of 125 cups of coffee instead of one cup of 125 g. Nutrient and energy intakes were computed by multiplying intakes by nutrient composition based on the Dutch food composition database (NEVO-table, 2016). AA composition was estimated by matching products with more than 1 g protein per 100 g product to similar products in the Danish Food Composition Table. For food items with less than 1 g protein per 100 g, all AA were set to zero. For Dutch items that did not have an equivalent product in the Danish Food Composition Table: (a) a ‘basic’ product was chosen based on the product’s largest protein source; (b) a comparable product was chosen to obtain the AA profile; (c) other databases were accessed to obtain the AA information; (d) a recipe was created; (e) or specific data for certain dairy products was obtained from Friesland Campina. Following the linking of all items, the AA profiles were adjusted for the quantity of protein present in Dutch products using the following formula: *mg amino acid = gram protein in NEVO × mg amino acid in Danish Food Composition Table*

### Laboratory analysis

#### Protein-bound AA in HM

For determination of BAA in HM samples, 0·20 ml of diluted hydrochloric acid containing 0·5 % 2-mercaptoethanol was added to the HM sample and mixed. Oxygen was removed by flushing the headspace of the tube with nitrogen for 60 s. Protein was hydrolysed by heating the mixture during 20–22 h. When the mixture was cooled down, 0·20 ml of sodium hydroxide solution, 2 ml demi water and 0·2 ml internal standard (Norvaline 20 ug) were added and mixed. The mixture was centrifuged, and a small part of the liquid was filtered over a 0·45 mm polyvinylidene difluoride filter. The peak area of each AA was compared with the peak area of the internal standard (Sigma).

#### Free AA in HM

To determine the free AA in HM, an ultra-fast liquid chromatography-based protocol was used. Each 50 ul milk sample was mixed with 1·0 ml internal standard solution (2·5 mg/ml L-norvaline). This mixture was centrifuged, and 25 ul of the supernatant was transferred into a sample vial. A pre-column derivatisation process was carried out by adding 30 ul of o-phthalaldehyde-reagent to the vial and mixing three times with a mixing volume of 45 ul. 1 ul of this o-phthalaldehyde-derivatised sample was injected and analysed in an ultra-fast liquid chromatography system with fluorimetry as detection. Standard AA solution Sigma AA-S-18 was used for calibration. To prepare the calibration AA solution, asparagine and tryptophan were added into Sigma AA-S-18 stock solution to reach a concentration of 2·5 uM/ml of each AA. Next, 0·50 ml, 1·00 ml, 2·00 ml and 5·00 ml of this solution were mixed with 1·6 ml of perchloric acid and further diluted to 50 ml. These calibration AA solutions were prepared to o-phthalaldehyde-derivate as described previously and measured in an ultra-fast liquid chromatography system. The calibration curve was constructed from peak areas and AA concentrations. Response factors of each AA were obtained by an extra analysis of standard AA solution containing internal standards.

### Statistical analysis

Participant characteristics were described as mean with sd or frequencies. To test differences in maternal-infant characteristics and stress measurements, unpaired student *t* tests and *χ*^*2*^ tests were used as appropriate. Participants were excluded from the final analyses when they had no data on AA dietary intake or HM AA composition. AA concentrations are reported as ug AA/ml sample. Data were checked for normal distribution and were log(10)-transformed if not normally distributed. Associations between maternal dietary AA intake and the AA composition of HM were tested using linear mixed models to be able to correct for within-person repeated measures. AA were tested as total AA, as separate individual AA and as total essential and total non-essential AA. As AA from one precursor family can be converted into other members of this family, AA were also grouped into precursor groups; the glutamate precursor group (glutamic acid, glutamine, arginine), the aspartate precursor group (aspartic acid, methionine isoleucine, threonine, lysine), the serine precursor group (serine, glycine), the pyruvate precursor group (valine, leucine, alanine), the aromatic precursor group (phenylalanine, tyrosine, tryptophan) and the histidine precursor group (histidine). Analyses were performed separately for the BAA and FAA. Considered covariates included gestational age at delivery, lactation stage, maternal BMI, being primiparous and smoking status^(1,3,4,10)^. Factors that influenced associations with a change in the regression coefficient of > 10 %, were considered relevant covariates and included in the final model. Gestational age at delivery and lactation stage were tested for effect modification with a *P*–_for interaction_ < 0·1 being considered significant. Given the relatively high number of associations studied, we corrected for multiple testing by means of the Bonferroni step-down (Holm) approach^(29)^. *P*-values are presented (1) crude, (2) adjusted for confounders and (3) adjusted for confounders and multiple testing. All statistical analyses were performed with the statistical software IBM SPSS version 26. A *P*-value (two-sided) of < 0·05 was considered statistically significant.

## Results

### Baseline characteristics

**Fig. 2.**
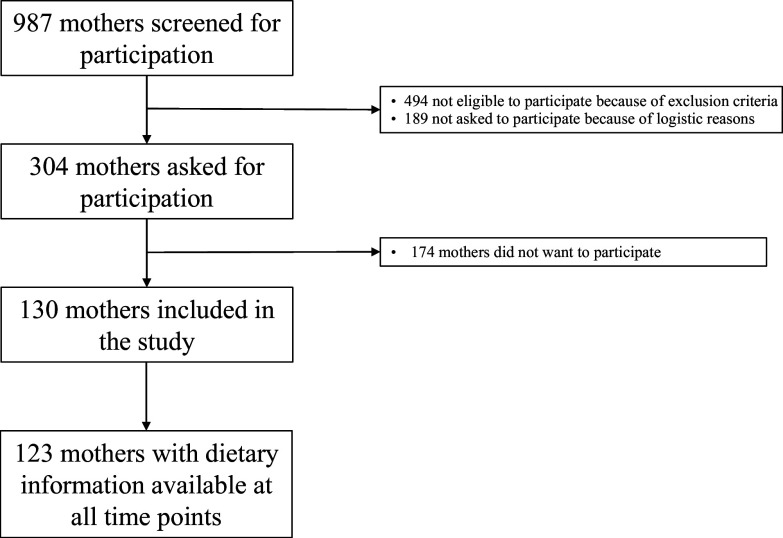
Flow chart of the study population.

In total, 123 women were eligible to participate and had data available for this study (see Fig. 2.) Mother and infant baseline characteristics are presented in Table 1a. Participants had a mean age of 32·4 (sd 3·8) years and mean BMI of 23·4 (sd 4·3). Their infants were born with a mean birth weight of 3112 (sd 980) g. Due to the study setup, the sample consisted of a relatively high percentage of mothers who gave birth prematurely (23 %). At p10, 87 % of participating mothers were exclusively breastfeeding, and this was 83 % at p24.

**Table 1a.** Maternal and infant characteristics (Mean values and standard deviations; numbers and percentages)

	*n* of participants with information on characteristic	Mean or *n*	sd or %
Maternal characteristics			
Age (years)			
Mean	118	32·4	
sd		3·8	
BMI (kg/m^2^)			
Mean	118	23·4	
sd		4·3	
Smoking	123		
Never		75	65·8 %
Past		1	0·9 %
Current		38	33·3 %
Alcohol intake	123		
Never		102	87·9 %
1/month		5	4·3 %
2–4/month		9	7·8 %
Parity	118		
Primiparous		74	60·2 %
Infant			
Birth weight (g)			
Mean	117	3112	
sd		980	
Gestational age at delivery	123		
Preterm^*^		28	22·8 %
Sex	123		
Male		60	48·8 %

*n*, number.

* < 37 weeks of gestation.

**Table 1b.** Maternal dietary energy, protein and amino acid intake as measured by the mean of three 24-h recall questionnaires (Mean values and standard deviations)

Intake component	Mean	sd
Energy total kcal	2055	575
Energy total kilojoule	8621	2412
Total protein (g/d)	73·9·	21·7
Plant protein (g/d)	33·5·	10·6
Animal protein (g/d)	40·4·	18·4
Intake specific amino acids (mg/d)		
Total	71 581	21 813
Essential	31 692	10 187
Non-essential	33 472	9953
Glutamate family	19 368	5651
Arginine	3911	1310
Glutamic acid	15 456	4593
Aspartate family	17 791	6036
Aspartic acid	5852	1971
Methionine	1583	545
Threonine	2617	853
Isoleucine	3279	1066
Lysine	4460	1698
Serine family	6491	1970
Serine	3585	1087
Glycine	2905	945
Pyruvate family	12 846	4055
Alanine	3192	1073
Valine	3977	1260
Leucine	5677	1787
Aromatic family	6759	2109
Phenylalanine	3400	1020
Tyrosine	2482	838
Tryptophan	878	268
Histidine family	1910	629
Histidine	1910	629

kcal, kilocalories; g, gram; mg, milligram; *n* 123.

### Maternal dietary intake and HM AA concentrations

Maternal dietary intake is shown in Table 1b indicating a mean energy intake of 2055 (sd 575) kcal/d. Protein accounts for 14 % of the total energy intake, with 55 % coming from animal-based sources and 45 % from plant-based sources. The most abundant AA in the participant’s diet were glutamic acid (15 456 (sd 4593) mg/d), aspartic acid (5852 (sd 1971) mg/d), leucine (5677 (sd 1787) mg/d), lysine (4460 (sd 1698) mg/d) and valine (3977 (sd 1260) mg/d). The lowest intakes were observed for methionine (1583 (sd 545) mg/d) and tryptophan (878 (sd 268) mg/d). Concentrations of BAA and FAA in HM samples are shown in online Supplementary Table 1. Mean BAA and essential BAA concentrations in the HM samples were 11 692, 10 363 and 9789 ug/ml (*P*–_for trend_ < 0·001) and 5986, 5343 and 4977 mg/ml on p10, p17 and p24, respectively. Mean FAA and essential FAA concentrations in HM samples were 256·9, 274·2 and 281·9 mg/ml (*P*±_for trend_ < 0·01) and 53·5, 50·3 and 45·0 mg/ml on p10, p17 and p24, respectively. The lowest BAA and FAA concentrations in HM were found for methionine, and the highest concentrations in HM were found for glutamine + glutamic acid.

### Association between maternal AA intake and AA concentrations in HM

Crude models did not show any associations between maternal AA intake and HM BAA composition, which did not change after correcting for confounding variables (Table 2). No effect modifiers were identified. Maternal AA intake of the glutamate family was positively associated with concentrations of glutamate family AA in HM (estimate of fixed effects: 0·002; *P* = 0·04), as was the intake of asparagine with asparagine concentrations in HM (estimate of fixed effects: 0·0002; *P* = 0·03) (Table 3). However, these associations disappeared after correction for parity and BMI. Maternal intake of arginine and lysine was negatively associated with its HM concentration, and intake of glutamine and threonine was positively associated with its HM concentrations, even after adjustment for parity and BMI (arginine: −0·0003; *P* = 0·01, lysine: −0·0004; *P* = 0·03, glutamate: 0·002; *P* = 0·03, threonine: 0·0008; *P* = 0·03). All associations disappeared after correction for multiple testing. No effect modifiers were identified.

**Table 2.** Association between maternal amino acid intake and the concentration of specific protein-bound amino acids in human milk (Crude and adjusted estimate and 95 % confidence intervals)

	Crude analysis				Analysis adjusted for covariates			
	Crude estimate	95 % CI	*P*	Corrected *P* ^*^	Adjusted estimate	95 % CI	*P*	Corrected *P* ^*^
Total BAA	–0·007	–0·02, 0·005	0·27	NS	–0·005	–0·02, 0·007	0·41	NS
Essential BAA	–0·007	–0·02, 0·008	0·33	NS	–0·006	–0·02, 0·009	0·45	NS
Non-essential BAA	–0·007	–0·02, 0·005	0·25	NS	–0·005	–0·02, 0·007	0·39	NS
Glutamate family	–0·001	–0·008, 0·006	0·79	NS	0·00003	–0·008, 0·008	0·99	NS
Glx	0·002	–0·005, 0·01	0·50	NS	0·003	–0·004, 0·01	0·42	NS
Arginine	–0·008	–0·02, 0·007	0·27	NS	–0·008	–0·02, 0·007	0·32	NS
Aspartate family	–0·006	–0·02, 0·008	0·39	NS	–0·006	–0·02, 0·007	0·46	NS
Asx	–0·009	–0·02, 0·006	0·24	NS	–0·009	–0·02, 0·09	0·27	NS
Methionine	–0·002	–0·01, 0·008	0·74	NS	–0·001	–0·01, 0·009	0·79	NS
Isoleucine	0·002	–0·01, 0·02	0·77	NS	0·004	–0·009, 0·02	0·54	NS
Threonine	–0·02	–0·04, 0·008	0·20	NS	–0·01	–0·04, 0·01	0·25	NS
Lysine	–0·006	–0·02, 0·009	0·43	NS	–0·006	–0·02, 0·01	0·47	NS
Serine family	–0·01	–0·03, 0·004	0·12	NS	–0·01	–0·03, 0·006	0·18	NS
Serine	–0·02	–0·04, 0·003	0·09	NS	–0·01	–0·04, 0·007	0·18	NS
Glycine	–0·01	–0·02, 0·005	0·19	NS	–0·009	–0·02, 0·005	0·21	NS
Pyruvate family	–0·009	–0·03, 0·008	0·32	NS	–0·006	–0·02, 0·01	0·47	NS
Valine	–0·009	–0·03, 0·007	0·29	NS	–0·007	–0·02, 0·01	0·42	NS
Leucine	–0·005	–0·02, 0·01	0·56	NS	–0·003	–0·02, 0·02	0·78	NS
Alanine	–0·01	–0·03, 0·006	0·22	NS	–0·01	–0·03, 0·008	0·28	NS
Aromatic family	–0·01	–0·03, 0·004	0·15	NS	–0·008	–0·02, 0·006	0·25	NS
Phenylalanine	–0·01	–0·03, 0·002	0·10	NS	–0·01	–0·03, 0·006	0·19	NS
Tyrosine	–0·009	–0·03, 0·007	0·26	NS	–0·007	–0·02, 0·009	0·39	NS
Histidine family	–0·008	–0·02, 0·007	0·29	NS	–0·007	–0·02, 0·007	0·33	NS
Histidine	–0·008	–0·02, 0·007	0·29	NS	–0·007	–0·02, 0·007	0·33	NS

BAA, protein-bound amino acids; Glx, sum of glutamic acid + glutamine; Asx, sum of aspartic acid + asparagine.

Associations are tested using linear mixed models. Crude and adjusted analysis are shown. Adjusted estimate is adjusted for maternal BMI, primiparity, maternal smoking and maternal alcohol consumption. *P*-values are shown before and after multiple testing correction. NS = not significant

* *P*-value corrected for multiple testing.

**Table 3.** Association between maternal amino acid intake and the concentration of specific free amino acids in human milk (Crude and adjusted estimate and 95 % confidence intervals)

	Crude analysis				Analysis adjusted for covariates			
	Crude estimate	95 % CI	*P*	Corrected *P* ^†^	Adjusted estimate	95 % CI	*P*	Corrected *P* ^†^
Total FAA	0·0004	–0·00007, 0·0009	0·09	NS	0·0003	–0·0002, 0·0008	0·19	NS
Essential FAA	–0·0001	–0·0003, 0·0001	0·34	NS	–0·0001	–0·0004, 0·0001	0·28	NS
Non-essential FAA	0·001	0·00002, 0·002	0·05	NS	0·0008	–0·0002, 0·002	0·10	NS
Glutamate family	0·002	0·00009, 0·003	0·04^*^	NS	0·001	–0·0001, 0·003	0·07	NS
Glx	0·003	0·0005, 0·005	0·02^*^	NS	0·002	0·0002, 0·005	0·03^*^	NS
Arginine	–0·0003	–0·0005, −0·00006	0·01^*^	NS	–0·0003	–0·0005, −0·00006	0·01^*^	NS
Aspartate family	0·00003	–0·0002, 0·0002	0·74	NS	0·00002	–0·0002, 0·0002	0·83	NS
Asx	0·0002	0·00002, 0·0004	0·03^*^	NS	0·0002	–0·00001, 0·0004	0·07	NS
Methionine	–0·0001	–0·0009, 0·0007	0·83	NS	–0·0001	–0·001, 0·0007	0·77	NS
Isoleucine	–0·0002	–0·0004, 0·00002	0·07	NS	–0·0002	–0·0004, 0·000007	0·06	NS
Threonine	0·0008	0·00009, 0·001	0·03^*^	NS	0·0008	0·00001, 0·002	0·03^*^	NS
Lysine	–0·0004	–0·0007, –0·00005	0·02^*^	NS	–0·0004	–0·0007, 0·00004	0·03^*^	NS
Serine family	–0·0002	–0·0006, 0·0002	0·43	NS	–0·0002	–0·0006, −0·0002	0·33	NS
Serine	–0·00001	–0·0005, 0·0004	0·83	NS	–0·0001	–0·0006, 0·0004	0·61	NS
Glycine	–0·0003	–0·0006, 0·00009	0·15	NS	–0·0002	–0·0006, 0·0001	0·17	NS
Pyruvate family	0·0001	–0·0003, 0·0005	0·60	NS	0·00002	–0·0004, 0·0004	0·90	NS
Valine	–0·00002	–0·0007, 0·0007	0·96	NS	–0·00007	–0·0008, 0·0006	0·84	NS
Leucine	–0·0002	–0·0005, 0·00008	0·15	NS	–0·0002	–0·0005, 0·00006	0·13	NS
Alanine	0·0005	–0·0003, 0·001	0·19	NS	0·0003	–0·0005, 0·001	0·41	NS
Aromatic family	–0·0001	–0·0004, 0·0002	0·39	NS	–0·0002	–0·0004, 0·0001	0·29	NS
Phenylalanine	–0·0002	–0·0004, 0·0001	0·26	NS	–0·0002	–0·0005, 0·0001	0·24	NS
Tyrosine	–0·0001	–0·0004, 0·0002	0·38	NS	–0·0001	–0·0004, 0·0001	0·31	NS
Tryptophan	0·0001	–0·001, 0·001	0·84	NS	–0·00003	–0·001, 0·001	0·96	NS
Histidine family	0·0002	–0·0002, 0·0006	0·29	NS	0·0002	–0·0003, 0·0006	0·43	NS
Histidine	0·0002	–0·0002, 0·0006	0·29	NS	0·0002	–0·0003, 0·0006	0·43	NS

FAA, free amino acids; Glx, sum of glutamic acid + glutamine; Asx, sum of aspartic acid + asparagine.

Associations are tested using linear mixed models. Crude and adjusted analysis are shown. The adjusted estimate is adjusted for maternal BMI. *P*-values are shown before and after multiple testing corrections.

* *P*-value significant at the 0·05 level.

† *P*-value corrected for multiple testing.

## Discussion

The results of this study suggest that there is no association between maternal AA intake and HM concentrations of BAA or FAA in the first month postpartum. Although intakes of glutamine, arginine, threonine and lysine were associated with their HM concentrations in free form, effect sizes were small, and the associations attenuated after adjustment for multiple testing, deeming these associations not clinically relevant.

HM BAA and FAA concentrations in our study were comparable to concentrations described previously in a systematic review with 3774 subjects^(4)^. The protein intake in our study population was sufficient according to the Dutch Dietary Reference Intakes for lactating women by the Health Council of the Netherlands^(30)^ and comparable to the intake of the general Dutch population^(31)^. To our knowledge, this is the first study examining the association between maternal intake of specific AA and their concentrations in milk. However, studies have been performed on the association between total protein intake and the AA content of HM. For example, Wurtman et al. showed that Guatemalan lactating women consuming a maize-based diet, which is typically low in total protein (mean 61 (sd 5) g/d) compared with women consuming an American diet (mean 85 (sd 5) g/d), had lower concentrations of total protein and most FAA^(15)^. Moreover, Villalpando et al. compared HM BAA between Mexican rural women consuming a maize-based diet (low in protein) and American lactating women consuming a Western-style diet (high in protein) and showed that some BAA, including serine, proline and cystine, were higher, while other BAA, including valine and isoleucine, were lower in milk of the Mexican women compared with American lactating women^(16)^. Four other studies did not observe associations between maternal protein intake and HM protein or AA concentrations^(19–22)^, consistent with our findings after corrections for multiple testing. Although differences in results may be related to differences in study designs, and rigour of information about dietary intake and analyses, it may also be postulated that it relates to the fact that lactating mothers in our study, in contrast to some of the previous studies, all had sufficient protein intakes within a certain range. It may be that maternal intake only affects HM AA composition if it is above or below certain levels.

The finding that maternal dietary AA intake is not associated with HM AA concentrations is interesting as previous research focusing on other HM components has shown associations between maternal dietary intake and its HM concentrations for several nutrients, particularly fatty acids^(11–13)^. Why some milk nutrients are, but some are not affected by the maternal diet is still not completely understood. It can be hypothesised that due to the importance of AA in almost all infant body processes, the HM AA concentrations are stable due to active transport or mammary gland synthesis^(32)^. In addition, concentrations of BAA in the maternal circulation seem to be remarkably constant, even under conditions of protein restriction^(33)^. Subsequently, since both BAA and FAA in HM can be transported from the maternal circulation and BAA can be synthesised out of FAA by the mammary gland itself^(32)^, this may explain the lack of association between maternal dietary AA intake and HM AA concentrations. However, the process behind the transportation of AA from the maternal bloodstream into the mammary gland and into HM is complex and not yet fully known^(32)^.

The strengths of this study are its longitudinal design and the timing and frequency of HM sample collection. Moreover, the first month postpartum is a sensitive time window, frequently missed in earlier HM research, in which breastfed infants are dependent on HM as their only source for nutrients. Therefore, knowledge of the factors influencing HM during these first weeks after birth is extremely important. Furthermore, we measured AA intake using 24-hR, which provides detailed and actual intake data. Moreover, the 24-hR were digital and self-administered, and this is assumed to be less burdensome for the participants and less biased by social desirable answering^(34)^. Limitations of this study are the relatively small sample size for an observational cohort study, which might hinder the generalisation of the results. Second, data on maternal diet were self-reported, which could have led to inaccurate reporting by the participants and therefore reporting bias. In addition, we used the mean of three 24-hR, whereas one could argue that FFQ are more suitable to assess habitual intake data^(35)^. However, as the intake of protein is rather stable over time, three 24-hR days are usually considered acceptable in the field^(36)^. In addition, AA intake was estimated based on food databases, which may not always reflect the exact AA composition of the foods consumed by the study participant. Moreover, the women in our cohort had protein and AA intakes within the normal range; therefore, it cannot be excluded that extremely high or low intakes would influence HM AA composition. Lastly, we performed a relatively strict multiple testing correction to prevent the likelihood of a type 1 error and false positive outcomes, which may have increased the likelihood of a false negative finding.

According to our results, there is no association between maternal intake of AA and their HM concentrations. However, future research is needed to further corroborate these findings and by exploring associations in specific vulnerable groups with extremely low or high protein/AA intakes. The results from this study highlight the importance of understanding the impact of dietary intake on HM composition and may serve (inter)national bodies in defining optimal dietary guidelines that can empower healthcare workers, dieticians and lactating mothers in making healthy dietary choices for both mother and child.

## Supporting information

Juncker et al. supplementary material Juncker et al. supplementary material

## Supplementary material

For supplementary material/s referred to in this article, please visit https://doi.org/10.1017/S0007114524001600.
