# Supplementary material for: Maternal dietary protein and amino acid intake is not associated with the amino acid composition of human milk in an affluent environment
Source: Br J Nutr. 2024 Sep 23;132(5):590–8. doi: 10.1017/S0007114524001600 (PMC11531934; doi:10.1017/S0007114524001600)
Supplement: Juncker et al. supplementary material [file S0007114524001600sup001.docx]

**Supplemental table 1**: concentrations of protein-bound and free amino acids in human milk over the study period

|  | **BAA in ug/L, mean (SD)** | | | **FAA in ug/L, mean (SD)** | | |
| --- | --- | --- | --- | --- | --- | --- |
|  | **p10** | **p17** | **p24** | **p10** | **p17** | **p24** |
| **Total** | 11848.6 (1886) | 10432.6 (1691) | 9798.3 (1757) | 302.0 (87.2) | 331.2 (79.3) | 335.5 (82.6) |
| **Essential** | 5993.6 (1061) | 5362.3 (952) | 4949.2 (1025) | 53.8 (21.8) | 50.6 (18.6) | 45.0 (13.1) |
| **Non-essential** | 5711.5 (827) | 5030.8 (742) | 4795.5 (751) | 214.2 (75.0) | 248.8 (71.4) | 261.1 (77.7) |
| **Glutamate family** | 2667.9 (360) | 2418.9 (258) | 2344.5 (300) | 172.5 (63.9) | 202.0 (63.5) | 215.4 (69.8) |
| Glx | 2233.4 (296) | 2015.2 (171) | 1963.2 (247) | 168.8 (65.4) | 198.7 (68.3) | 211.9 (74.1) |
| Arginine | 434.5 (128) | 403.7 (115) | 381.3 (126) | 3.6 (2.4) | 3.3 (1.5) | 3.5 (1.7) |
| **Aspartate family** | 3230.0 (534) | 2753.7 (501) | 2624 (504) | 21.4 (9.2) | 26.0 (11.3) | 21.6 (6.2) |
| Asx | 1383.2 (211) | 1154.6 (219) | 1102.3 (202) | 5.2 (3.1) | 6.1 (2.9) | 6.2 (3.2) |
| Methionine | 207.5 (113) | 185.5 (37) | 160.0 (33) | 1.2 (0.7) | 4.8 (6.3) | 1.3 (1.1) |
| Isoleucine | 668.3 (111) | 612.9 (95) | 563.8 (90) | 2.2 (1.8) | 2.0 (2.0) | 1.9 (1.0) |
| Threonine | 652.2 (130) | 578.6 (116) | 543.7 (132) | 9.6 (5.0) | 10.9 (5.7) | 8.9 (3.3) |
| Lysine | 987.1 (175) | 835.0 (147) | 817.9 (167) | 5.4 (4.5) | 4.2 (3.0) | 5.2 (3.3) |
| **Serine family** | 1080.1 (234.9) | 957.7 (216) | 887.1 (235) | 15.5 (6.4) | 18.1 (4.9) | 18.2 (6.1) |
| Serine | 716.4 (151) | 637.8 (138) | 588.1 (150) | 9.5 (4.3) | 11.0 (3.5) | 10.7 (4.3) |
| Glycine | 363.7 (86) | 319.9 (80) | 299.0 (86) | 6.0 (2.5) | 7.2 (2.0) | 7.6 (2.5) |
| **Pyruvate family** | 3386.6 (575) | 3051.1 (513) | 2786.8 (558) | 43.4 (14.2) | 33.6 (12.9) | 31.9 (8.6) |
| Valine | 755.9 (151) | 700.1 (128) | 600.0 (130) | 19.1 (5.2) | 7.9 (8.0) | 6.7 (2.0) |
| Leucine | 1379.3 (220) | 1239.1 (199) | 1130.6 (270) | 4.3 (4.4) | 4.6 (5.3) | 4.7 (2.6) |
| Alanine | 563.2 (121) | 498.4 (109) | 472.3 (117) | 17.8 (6.7) | 19.0 (5.0) | 18.6 (5.3) |
| **Aromatic family** | 1019.4 (207) | 910.8 (182) | 838.3 (196) | 8.0 (4.6) | 11.4 (3.8) | 11.3 (3.8) |
| Phenyalanine | 567.8 (113) | 505.8 (103) | 467.6 (109) | 3.1 (1.9) | 3.3 (2.6) | 2.5 (2.0) |
| Tyrosine | 451.6 (101) | 404.9 (81) | 370.6 (88) | 3.7 (2.3) | 3.7 (2.1) | 3.1 (1.3) |
| Tryptophan | - | - | - | 1.2 (1.6) | 4.5 (3.0) | 5.6 (1.6) |
| **Histidine family** | 341.0 (62) | 300.9 (56) | 284.1 (57) | 4.1 (1.7) | 5.1 (1.7) | 4.6 (2.1) |
| Histidine | 341.0 (62) | 300.9 (56) | 284.1 (57) | 4.1 (1.7) | 5.1 (1.7) | 4.6 (2.1) |

Abbreviations: BAA, protein-bound amino acids. ug/L, microgram per liter. SD, standard deviation. FAA, free amino acids. p, post-partum day. Glx, sum of glutamic acid + glutamine. Asx, sum of aspartic acid + asparagine.

Protein-bound tryptophan was not measured in the human milk samples.
